# Optimal two-stage group sequential designs based on Mann-Whitney-Wilcoxon test

**DOI:** 10.1371/journal.pone.0318211

**Published:** 2025-02-20

**Authors:** Yeonhee Park

**Affiliations:** 1 Department of Statistics, Sungkyunkwan University, Seoul, South Korea; Ege University, Faculty of Medicine, TÜRKIYE

## Abstract

The Mann-Whitney-Wilcoxon test, often referred to as the Mann-Whitney U test or Wilcoxon rank-sum test, is a non-parametric statistical test used to compare two independent groups when the dependent variable is ordinal or continuous but not normally distributed. It’s particularly useful for small sample sizes or when the assumptions of parametric tests, such as the t-test, are violated, including cases where the data is skewed. This study focuses on the Mann-Whitney-Wilcoxon test for ordinal data, which frequently arises in biomedical research when the proportional odds assumption does not hold. Currently, there are no optimal two-stage randomized clinical trial designs utilizing the Mann-Whitney-Wilcoxon test. To address this research gap, our study proposes optimal two-stage designs based on the Mann-Whitney-Wilcoxon test. We demonstrate the application of these designs through illustrative examples and evaluate their operating characteristics.

## Introduction

Outcomes in clinical trials are often ordinal. In 2020, the World Health Organization (WHO) developed a nine-point clinical progression scale to track COVID-19 severity over time [1]. The scale ranges from 0 (no infection) to 8 (death), covering the full spectrum of clinical outcomes. This comprehensive scale has been widely used in COVID-19 trials and cohort studies [2–4]. Also, the modified Rankin Score (mRS) is a measure of disability ranging from 0 (no symptoms) to 6 (death). This is a widely used outcome measure in stroke clinical trials [5–7]. For example, randomized clinical trials such as NCT05199194, NCT05046106, and NCT00059332 primarily measure the mRS to evaluate the effectiveness of the intervention.

In practice, a dichotomized approach to ordered categorical variables is often used for comparative tests. For example, dichotomized mRS (0–1/2–6, 0–2/3–6, and 0–4/5–6) is commonly used [8–10]. However, as Altman and Royston (2006) [11] pointed out, dichotomizing leads to a reduction in statistical power to detect relevant treatment effects [12, 13]. To address the issue of analyzing ordinal endpoints, proportional odds logistic regression has been recommended. The proportional odds assumption states that the relationship between the independent variables and the log-odds of the cumulative probabilities of the ordinal categories is the same across all levels or categories of the dependent variable. Using the proportional odds model, Whitehead (1993) [14] proposes a statistical method to calculate the sample size based on the score test statistic for two-arm randomized clinical trials. The method by Whitehead (1993) [14] was improved by calculating higher order moments of the test statistic using the Cornish-Fisher series and the Edgeworth series [15]. These proportional odds methods preserve the ordinal nature of outcome measures, but the proportional odds assumption can be violated in clinical trials. As an alternative to avoid the proportional odds assumption, the Mann-Whitney-Wilcoxon test or other linear rank tests and win odds test are utilized. Shuster et al. (2002) [16] illustrates a minimax two-stage design using the calculation for a single-stage design and variances of the test statistics at the first and second stages. Shuster et al. (2004) [17] and Nowak et al. (2022) [18] investigate the Mann-Whitney-Wilcoxon test in the context of group sequential trials using the asymptotic canonical joint distribution. Hilton and Mehta (1993) [19] and Rabbee et al. (2003) [20] propose methods of sample size calculation applicable to any linear rank test under a general stochastically ordered alternative hypothesis for single-stage clinical trials. Moreover, Gasparyan et al. (2021) [21] proposes a sample size calculation based on the single-stage win odds test, which is used to analyze hierarchical composite endpoints, for ordinal variables.

Currently, no optimal two-stage randomized clinical trial designs utilize the Mann-Whitney-Wilcoxon test. Optimal two-stage designs based on the Mann-Whitney-Wilcoxon test can offer several advantages, particularly in terms of efficiency and flexibility [22–25]. These designs can reduce the total sample size needed to reach conclusive results, as unpromising trials can be stopped early, conserving resources and time. This flexibility not only protects participants from ineffective treatments but also minimizes patient exposure to potentially harmful interventions.

To fill this gap, we propose new methods to determine the sample size and monitoring rule for the optimal two-stage randomized clinical trial designs based on the Mann-Whitney-Wilcoxon test. We provide two types of the two-stage designs called F design and FS design allowing early stopping of the trial due to futility or superiority of the experimental treatment. In addition, we provide three criteria to select the optimal designs that minimize the expected total sample size. These methodologies offer clinical practitioners user-friendly study design options, enabling them to easily identify appropriate designs tailored to their specific study objectives.

The rest of this paper is organized as follows. We first propose optimal two-stage designs using the Mann-Whitney-Wilcoxon test. We provide explicit formulas for calculating sample sizes and identifying monitoring rules based on the optimal design parameters. We illustrate the methods with two examples and compare the designs. Additionally, we apply the proposed methods to redesign a trial and provide concluding remarks.

## Optimal two-stage designs using Mann-Whitney-Wilcoxon test

We consider two-arm clinical trials in which patients are enrolled and randomized with a 1:1 ratio to either a control *C* or an experimental treatment *E*. Let *X* be a treatment assignment having 1 or 2 for the patient receiving treatment *C* or *E*, respectively, and *Y * be the ordinal outcome with *J* ordered levels, i.e., *y* ∈ { 1 , 2 , … , *J* } . Without loss of generality, we regard the smallest outcome *Y* = 1 as the best clinical outcome and the largest outcome *Y* = *J* as the worst clinical outcome. For *i* = 1 , 2 and *j* = 1 , 2 , … , *J*, let $p_{ij}=\mathrm{Pr}(Y=j|X=i)$ be the probability that an ordinal outcome on treatment *i* falls in the *j*th level. Let *F* and *G* be the distribution function of *Y * on treatment *C* and *E*, respectively. Suppose that we are interested in testing the null hypothesis $H_{0}:F(y)=G(y)$ versus the alternative hypothesis $H_{a}:F(y)<G(y)$. The Mann-Whitney-Wilcoxon test is used to compare two independent samples based on the ranks of the observed ordinal outcome data. Specifically, let $Y_{1}\sim F$ and $Y_{2}\sim G$ denote the two independent random variables. Since the smallest value indicates the best outcome, we can compare the outcomes between two arms: for any *l* , *m* = 1 , 2 , … ,  the event of $Y_{1l}>Y_{2m}$ indicates that *E* wins while *C* loses; the event $Y_{1l}<Y_{2m}$ indicates that *C* wins while *E* loses; the event $Y_{1l}=Y_{2m}$ indicates that *C* and *E* are equivalent. This constructs the test statistics as follows. Based on the samples $Y_{11},\ldots ,Y_{1n_{1}}$ from *F* and $Y_{21},\ldots ,Y_{2n_{2}}$ from *G*, the Mann-Whitney-Wilcoxon test statistic is defined in [26] and [17] by


$$\begin{array}{c} W=\overset{n_{1}}{\underset{l=1}{\sum }}\overset{n_{2}}{\underset{m=1}{\sum }}\{I(Y_{1l}>Y_{2m})-I(Y_{1l}<Y_{2m})\}∕(n_{1}n_{2}). \end{array}$$ (1)

This accounts for the proportion of all comparisons leading to a win for arm *E*, i.e., *F* ( *y* ) < *G* ( *y* ) . In the context of the clinical trials investigating the treatment efficacy of arms *C* and *E*, ordinal data measure the treatment efficacy, and the event that *E* wins corresponds to the superiority of the experimental drug *E* while the event that *C* wins corresponds to the futility of the experimental drug *E*. We use the Mann-Whitney-Wilcoxon test based on (1) to propose an optimal two-stage design for ordinal outcomes.

Before we see the optimal two-stage design for ordinal outcomes based on the Mann-Whitney-Wilcoxon test (1), we first review the Mann-Whitney-Wilcoxon test for the design. For *i* = 1 , 2, *j* = 1 , 2 , … , *J*, and *k* = 1 , 2, let $n_{ijk}$ denote the corresponding observed frequencies for the *j*th level at the *k*th analysis when treatment *i* is received. Then, $n_{\cdot jk}=n_{1jk}+n_{2jk}$ denotes the marginal frequency for the *j*th level at the *k*th analysis. Based on the accumulating data at the *k*th analysis, (1) becomes


$$\begin{array}{c} W_{k}=[\overset{J}{\underset{j=2}{\sum }}n_{1jk}(\overset{j-1}{\underset{l=1}{\sum }}n_{2lk})-\overset{J}{\underset{j=2}{\sum }}n_{2jk}(\overset{j-1}{\underset{l=1}{\sum }}n_{1lk})]∕(\overset{J}{\underset{j=1}{\sum }}n_{1jk}\overset{J}{\underset{j=1}{\sum }}n_{2jk}), \end{array}$$ (2)

which is the Mann-Whitney-Wilcoxon test statistic used at the *k*th analysis. The test statistic $W_{k}$ is standardized to


$$\begin{array}{c} T_{k}=W_{k}∕\sqrt{\frac{\underset{j}{\sum }n_{\cdot jk}+1}{3\underset{j}{\sum }n_{1jk}\underset{j}{\sum }n_{2jk}}\{1-\frac{\underset{j}{\sum }(n_{\cdot jk}^{3}-n_{\cdot jk})}{{\left(\underset{j}{\sum }n_{\cdot jk}\right)}^{3}-\underset{j}{\sum }n_{\cdot jk}}\}}, \end{array}$$ (3)

where the denominator of (3) is the estimate of standard deviation of $W_{k}$ under the null hypothesis [17]. For a large sample, Shuster et al. (2004) [17] provides the variance of $W_{k}$ approximated by $(Q+R)∕n_{k}$, where


$$p^{+}=\overset{J-1}{\underset{j=1}{\sum }}p_{2j}\overset{J}{\underset{l=j+1}{\sum }}p_{1l},\;p^{-}=\overset{J-1}{\underset{j=1}{\sum }}p_{1j}\overset{J}{\underset{l=j+1}{\sum }}p_{2l},\;D=p^{+}-p^{-},$$

$$Q=\overset{J-1}{\underset{j=1}{\sum }}p_{1j}(\overset{J}{\underset{l=j+1}{\sum }}p_{2l}{)}^{2}+\overset{J}{\underset{j=2}{\sum }}p_{1j}(\overset{j-1}{\underset{l=1}{\sum }}p_{2l}{)}^{2}-2\overset{J-1}{\underset{j=2}{\sum }}p_{1j}(\overset{j-1}{\underset{l=1}{\sum }}p_{2l})(\overset{J}{\underset{l=j+1}{\sum }}p_{2l})-D^{2},$$

and


$$R=\overset{J-1}{\underset{j=1}{\sum }}p_{2j}(\overset{J}{\underset{l=j+1}{\sum }}p_{1l}{)}^{2}+\overset{J}{\underset{j=2}{\sum }}p_{2j}(\overset{j-1}{\underset{l=1}{\sum }}p_{1l}{)}^{2}-2\overset{J-1}{\underset{j=2}{\sum }}p_{2j}(\overset{j-1}{\underset{l=1}{\sum }}p_{1l})(\overset{J}{\underset{l=j+1}{\sum }}p_{1l})-D^{2}.$$

We notice that $D_{0}$, which indicates the value of *D* under the null hypothesis, is zero. The quantity of *D* under the alternative hypothesis is denoted by $D_{a}$. To distinguish the statistics under the null and alternative hypotheses, hereafter we will use notations $Q_{0}$ and $R_{0}$ for *Q* and *R*, respectively, under the null hypothesis and $Q_{a}$ and $R_{a}$ for *Q* and *R*, respectively, under the alternative hypothesis.

### Sample size calculation for two-stage design using Mann-Whitney-Wilcoxon test

The two-stage design consists of an interim analysis planned when the total accrual reaches $n_{1}$ patients per group, i.e., $n_{1}=\sum_{j=1}^{J}n_{1j1}=\sum_{j=1}^{J}n_{2j1}$, and the final analysis is performed after the evaluation of total planned number of $n_{2}$ patients per group, i.e., $n_{2}=\sum_{j=1}^{J}n_{1j2}=\sum_{j=1}^{J}n_{2j2}$. At the interim, we monitor the treatment efficacy based on the accumulating data and determine whether to continue or stop the trial.

#### Futility monitoring design (F design).

When the total accrual reaches $2n_{1}$ patients, an interim analysis is performed to test for futility, such that the trial stops if the experimental treatment *E* is more futile than the control *C*, i.e., $W_{1}\leq t_{1}$ for a prespecified constant $t_{1}$. If $W_{1}>t_{1}$, we continue to enroll $2(n_{2}\;-\;n_{1})$ more patients for the second stage, and the test proceeds to the final analysis. Based on all enrolled patients in the trial, if $W_{2}>t_{2}$ for a prespecified constant $t_{2}$, the test terminates with the rejection of the null hypothesis.

Let $\alpha_{1}$ and $\beta_{1}$ be given such that $1-\alpha_{1}$ and $1-\beta_{1}$ denote the probability of making a correct decision at the interim analysis under the null hypothesis and the alternative hypothesis, respectively. By the definition of $\alpha_{1}$, i.e., $\mathrm{Pr}(W_{1}\leq t_{1}|H_{0})=1-\alpha_{1}$, we obtain


$$\begin{array}{c} t_{1}=z_{\alpha_{1}}\sqrt{\frac{Q_{0}+R_{0}}{n_{1}}} \end{array}$$ (4)

where $z_{\alpha_{1}}$ denotes the critical value of the standard normal distribution at $\alpha_{1}$. With the Eq (4), the definition of $\beta_{1}$, i.e., $\mathrm{Pr}(W_{1}>t_{1}|H_{a})=1-\beta_{1}$, gives


$$\begin{array}{c} n_{1}=(\frac{z_{\alpha_{1}}\sqrt{Q_{0}+R_{0}}+z_{\beta_{1}}\sqrt{Q_{a}+R_{a}}}{D_{a}}{)}^{2}. \end{array}$$ (5)

Assuming that the type I error rate is at most *α* and the expected power is at least 1 - *β*, we set $\mathrm{Pr}(W_{2}>t_{2}|H_{0})=\alpha$ and $\mathrm{Pr}(W_{2}>t_{2}|H_{a})-\mathrm{Pr}(W_{1}\leq t_{1}|H_{a})=1-\beta$, because $\mathrm{Pr}(W_{2}>t_{2},W_{1}>t_{1}|H_{0})\leq \mathrm{Pr}(W_{2}>t_{2}|H_{0})$ and $\mathrm{Pr}(W_{2}>t_{2}|H_{a})\;-\;\mathrm{Pr}(W_{1}\leq t_{1}|H_{a})\leq \mathrm{Pr}(W_{2}>t_{2},W_{1}>t_{1}|H_{a})$. Therefore, we obtain


$$\begin{array}{c} t_{2}=z_{\alpha }\sqrt{\frac{Q_{0}+R_{0}}{n_{2}}} \end{array}$$ (6)

and


$$\begin{array}{c} n_{2}=(\frac{z_{\alpha }\sqrt{Q_{0}+R_{0}}+z_{\beta_{2}}\sqrt{Q_{a}+R_{a}}}{D_{a}}{)}^{2}, \end{array}$$ (7)

where $\beta_{2}=\beta -\beta_{1}$.

#### Futility and superiority monitoring design (FS design).

The interim analysis can be conducted to evaluate the efficacy of the intervention and to stop the trial if an experimental treatment is ineffective or is found to be significantly more effective than the control. Let $v_{1}$, $v_{2}$, and $v_{3}$ be the prespecified thresholds for the analyses. Specifically, we stop the trial for futility if $W_{1}\leq v_{1}$, and we stop the trial for superiority if $W_{1}>v_{2}$. Otherwise, i.e., $v_{1}<W_{1}\leq v_{2}$, we continue to enroll $2(n_{2}-n_{1})$ more patients for the second stage. At the final analysis based on all $2n_{2}$ enrolled patients, we reject the null hypothesis if $W_{2}>v_{3}$.

Let $\alpha_{1}$ and $\beta_{2}$ be given such that $1-\alpha_{1}$ and $1-\beta_{2}$ denote the probability of making a correct decision at the interim analysis under the null hypothesis and the alternative hypothesis, respectively, i.e., $\mathrm{Pr}(W_{1}\leq v_{1}|H_{0})=1-\alpha_{1}$ and $\mathrm{Pr}(W_{1}>v_{2}|H_{a})=1-\beta_{2}$. Let $\alpha_{2}=\mathrm{Pr}(W_{1}>v_{2}|H_{0})$ and $\beta_{1}=\mathrm{Pr}(W_{1}\leq v_{1}|H_{a})$. By the definition of $\alpha_{1}$, $\alpha_{2}$, and $\beta_{1}$, we have


$$\begin{array}{c} v_{1}=z_{\alpha_{1}}\sqrt{\frac{Q_{0}+R_{0}}{n_{1}}}, \end{array}$$ (8)

$$\begin{array}{c} v_{2}=z_{\alpha_{2}}\sqrt{\frac{Q_{0}+R_{0}}{n_{1}}}, \end{array}$$ (9)

and


$$\begin{array}{c} n_{1}=(\frac{z_{\alpha_{1}}\sqrt{Q_{0}+R_{0}}-z_{1-\beta_{1}}\sqrt{Q_{a}+R_{a}}}{D_{a}}{)}^{2}. \end{array}$$ (10)

We notice that the overall type I error rate is $\mathrm{Pr}(W_{1}>v_{2}|H_{0})\;+\;\mathrm{Pr}(v_{1}<W_{1}\leq v_{2},W_{2}>v_{3}|H_{0})$, which is less than or equal to $\mathrm{Pr}(W_{1}>v_{2}|H_{0})\;+\;\mathrm{Pr}(W_{2}>v_{3}|H_{0})$. Since we want the type I error rate to be at most *α*, we set $\mathrm{Pr}(W_{2}>v_{3}|H_{0})$ to be $\alpha \;-\;\alpha_{2}$. In addition, we observe that


$$\begin{array}{cccc} \text{power} & = & \mathrm{Pr}(W_{1}>v_{2}|H_{a})+\mathrm{Pr}(v_{1}<W_{1}\leq v_{2},W_{2}>v_{3}|H_{a}) & \\ & \geq & 1-\beta_{2}+\mathrm{Pr}(v_{1}<W_{1}\leq v_{2}|H_{a})+\mathrm{Pr}(W_{2}>v_{3}|H_{a})-1=\mathrm{Pr}(W_{2}>v_{3}|H_{a})-\beta_{1}. & \end{array}$$

Since we want the expected power to be at least 1 - *β*, we set $\mathrm{Pr}(W_{2}>v_{3}|H_{a})$ to be $1-(\beta -\beta_{1})$. Therefore, we obtain


$$\begin{array}{c} v_{3}=z_{\alpha -\alpha_{2}}\sqrt{\frac{Q_{0}+R_{0}}{n_{2}}} \end{array}$$ (11)

and


$$\begin{array}{c} n_{2}=(\frac{z_{\alpha -\alpha_{2}}\sqrt{Q_{0}+R_{0}}-z_{1-(\beta -\beta_{1})}\sqrt{Q_{a}+R_{a}}}{D_{a}}{)}^{2}. \end{array}$$ (12)

### Optimal choice of design parameters

Determination of the thresholds and sample sizes for a two-stage design requires the specification of the design parameters such as $\alpha_{1}$ and $\beta_{1}$ for *F* design and $\alpha_{1}$, $\alpha_{2}$, and $\beta_{1}$ for FS design. We consider three criteria to specify the design parameters: (1) minimizing the expected total sample size under the null hypothesis, (2) minimizing the expected total sample size under the alternative hypothesis, and (3) minimizing the expected overall total sample size. The overall total sample size is defined as the total sample size, averaged equally across the null and alternative hypotheses.

In two-stage designs with futility monitoring (i.e., F design), if $W_{1}\geq t_{1}$, we stop the trial for futility, and the total sample size is $2n_{1}$. Otherwise, i.e., $W_{1}<t_{1}$, the trial continues to enroll $2(n_{2}-n_{1})$ patients for the second stage, and the total sample size is $2n_{2}$. Therefore, the expected total sample size under the null hypothesis is $2n_{1}+2(n_{2}-n_{1})\alpha_{1}$, the expected total sample size under the alternative hypothesis is $2n_{1}+2(n_{2}-n_{1})(1-\beta_{1})$, and the expected overall total sample size is $2n_{1}+(n_{2}-n_{1})(\alpha_{1}+1-\beta_{1})$. Of note, $n_{1}$ and $n_{2}$ are functions of $\alpha_{1}$ and $\beta_{1}$. We choose the optimal values of $\alpha_{1}$ and $\beta_{1}$ according to the criteria over $\alpha \leq \alpha_{1}$ and $\beta ∕2\leq \beta_{1}<\beta$. The constraint of $\beta ∕2\leq \beta_{1}$ guarantees $n_{1}\leq n_{2}$.

Similarly, in two-stage designs with futility and superiority monitoring (i.e., FS design), the expected total sample size under the null hypothesis is $2n_{1}+2(n_{2}-n_{1})(\alpha_{1}-\alpha_{2})$, the expected total sample size under the alternative hypothesis is $2n_{1}+2(n_{2}-n_{1})(\beta_{2}-\beta_{1})$, and the expected overall total sample size is $2n_{1}+(n_{2}-n_{1})(\alpha_{1}-\alpha_{2}+\beta_{2}-\beta_{1})$. The values of $\beta_{2}$, $n_{1}$, and $n_{2}$ depend on $\alpha_{1},\alpha_{2}$, and $\beta_{1}$, and the optimal values of $\alpha_{1}$, $\alpha_{2}$, and $\beta_{1}$ are chosen according to the criteria over $\alpha \leq \alpha_{1}<1$, $0<\alpha_{2}<\alpha$, and $0<\beta_{1}<\beta$. Any pairs of $\alpha_{1}$, $\alpha_{2}$, and $\beta_{1}$ are excluded unless $0<n_{1}<n_{2}$.

We find that the minimax criterion is not applicable to determine the design parameters of the two-stage designs. In F design, minimizing the maximum sample size is equivalent to maximizing $\beta_{1}$ over $\beta ∕2\leq \beta_{1}<\beta$. Thus, it chooses the lower boundary of $\beta_{1}$ as the optimal value to attain the minimum value of $n_{2}$ regardless of $\alpha_{1}$. Thus, the value of $\alpha_{1}$ cannot be uniquely determined for F design. Similarly, in FS design, the minimum value of $n_{2}$ is attained over $0<\alpha_{2}<\alpha$ and $0<\beta_{1}<\beta$ satisfying $0<n_{1}<n_{2}$, but the value of $\alpha_{1}$ is not uniquely determined.

### Decision rule for single-stage design

The decision rule for a single-stage design can be straightforwardly derived from that of a two-stage design. By setting $t_{1}=-\infty$, it becomes impossible to stop the trial for futility at stage 1. Furthermore, by setting $n_{1}=n_{2}$, the second stage sample size is effectively eliminated, as the entire sample is enrolled in a single stage. This simplifies the design, removing the need for interim analysis or additional patient accrual after the initial enrollment. Specifically, the single-stage design employs the Mann-Whitney-Wilcoxon test statistic denoted by *W*, calculated using data from all 2*n* patients at the end of the trial. The null hypothesis is rejected if *W* > *t*, where *t* is a prespecified threshold.

The decision rule  ( *t* , *n* )  of the single–stage design is determined to ensure that the design satisfies $\mathrm{Pr}(W_{2}>t_{2}|H_{0})=\alpha$ and $\mathrm{Pr}(W_{2}>t_{2}|H_{a})=1-\beta$, meeting the specified type I error rate and power requirements. Similarly to the derivation of Eqs (4)–(12), by using the property of the Mann-Whitney-Wilcoxon test statistic [17], we obtain


$$t=z_{\alpha }\sqrt{\frac{Q_{0}+R_{0}}{n}}\;\text{ and }\;n=(\frac{z_{\alpha }\sqrt{Q_{0}+R_{0}}+z_{\beta }\sqrt{Q_{a}+R_{a}}}{D_{a}}{)}^{2}.$$

### Examples

#### Example 1.

We consider a clinical trial whose overall clinical outcome is ordinal with 3 categories and a descending order of desirability, e.g., 1=clinical benefit without adverse effects (AEs), 2=clinical benefit with some AEs, and 3=survival without clinical benefit but with AEs. Suppose that the distribution of the desirability score for the standard treatment is uniform, i.e., the probabilities of each category for the standard treatment are 1 ∕ 3, while investigators wish to have the probabilities 1 ∕ 2, 1 ∕ 3, and 1 ∕ 6 for each category for the experimental treatment. Target error rates are set to be *α* = 0 . 05 and *β* = 0 . 2 for the test comparing two treatments.

First, we consider a two-stage design with futility monitoring. By applying the Eqs (4)–(7), the optimal decision rule $\left(t_{1},n_{1},t_{2},n_{2}\right)$ is determined according to the criterion. Let EN0 denote the expected total sample size under the null hypothesis, and ENa denote the expected total sample size under the alternative hypothesis. As described, Criterion 1 is to minimize EN0, Criterion 2 is to minimize ENa, and Criterion 3 is to minimize (1/2)EN0 + (1/2)ENa. Second, we consider a two-stage design monitoring both futility and superiority. Similarly to the design with futility monitoring, by applying the Eqs (8)–(12), the decision rule $\left(v_{1},v_{2},n_{1},v_{3},n_{2}\right)$ is determined according to the criterion. Lastly, we include a single-stage design, referred to as *1 design*, to provide a basis for comparison. For consistency in notation, the decision rule  ( *t* , *n* )  is represented in the two–stage format as $\left(t_{1},n_{1},t_{2},n_{2})=(-\infty ,n,t,n\right)$, where $t_{1}=-\infty$ and $n_{1}=n_{2}=n$. Table 1 summarizes the decision rules of the designs.

**Table 1:** Decision rule and its operating characteristics such as overall type I error rate denoted by $\hat{\alpha }$, power, expected sample size under null hypothesis denoted by EN0, and expected sample size under alternative hypothesis denoted by ENa. The sample sizes $n_{1}$ and $n_{2}$ are obtained by rounding up the results.

		Decision rule					Operating characteristics			
		$t_{1}$		$n_{1}$	$t_{2}$	$n_{2}$	$\hat{\alpha }$	Power	EN0	ENa
F design	Criterion 1	0.060		32	0.125	104	0.040	0.836	113.64	191.47
F design	Criterion 2	-36.75		1	0.127	100	0.049	0.899	200	200
F design	Criterion 3	0.047		30	0.127	100	0.040	0.833	110.51	185.66
		Decision rule					Operating characteristics			
		$v_{1}$	$v_{2}$	$n_{1}$	$v_{3}$	$n_{2}$	$\hat{\alpha }$	Power	EN0	ENa
FS design	Criterion 1	0.061	0.423	32	0.125	105	0.042	0.834	112.22	184.68
FS design	Criterion 2	-0.046	0.270	42	0.151	83	0.043	0.817	135.95	137.64
FS design	Criterion 3	-0.004	0.314	38	0.144	83	0.043	0.825	121.44	143.73
		Decision rule					Operating characteristics			
		$t_{1}$		$n_{1}$	$t_{2}$	$n_{2}$	$\hat{\alpha }$	Power	EN0	ENa
1 design		-*∞*		73	0.149	73	0.048	0.799	146	146

To investigate the operating characteristics of the proposed designs, we conduct a simulation study with 10000 replications. We report four performance metrics: overall type I error rate denoted by $\hat{\alpha }$, power (i.e., one minus overall type II error rate), and the expected sample size under null and alternative hypotheses denoted by EN0 and ENa, respectively. For all three criteria, all designs preserve overall type I and II error rates at the target rates. The overall type I error rates are slightly smaller than the nominal level, which yet does not sacrifice the power, i.e., overall type II error rates are also smaller than the nominal level. Criterion 1 yields a smaller size of the first stage for both F design and FS design compared to other criteria, which leads to a smaller expected sample size under the null hypothesis. Specifically, both the F design and FS design with Criterion 1 spend only 30% of information of the trial to make the decision earlier than others. The FS design with Criterion 2 or Criterion 3 requires $n_{2}=$83 patients per group, which is the smallest maximum sample size of the trial among all six decision rules. We also notice that the the F design with Criterion 2 uses extremely small threshold for futility monitoring, and $n_{1}=1$ is calculated. With $n_{1}=1$ being calculated, implementing a two-stage design becomes impractical and is therefore not recommended. While 1 design requires a smaller maximum sample size compared to the two-stage designs (F and FS designs), we observe that two-stage designs—except for the F design with Criterion 2—tend to use a smaller expected sample size under the null hypothesis. This is an advantage, as it reduces the number of patients enrolled when the treatment shows no effect, thus saving resources and minimizing patient exposure to potentially ineffective treatments. Moreover, most two-stage designs utilize a larger expected sample size under the alternative hypothesis, which is advantageous. Enrolling more patients in the effective treatment not only enhances the precision of estimating the treatment effect but also maximizes the potential benefits for participants receiving the treatment.

#### Example 2.

We consider an example trial illustrated in [14], whose primary endpoint is the patients’ response evaluating the progress after the treatment, which is categorized as *very good* (=1), *good* (=2), *moderate* (=3), or *poor* (=4). Let $p_{ij},i=1,2,j=1,2,3,4$ denote the probability that an ordinal outcome on treatment *i* falls in the *j*th level. Suppose that we have $p_{11}=0.2,p_{12}=0.5,p_{13}=0.2$, and $p_{14}=0.1$ for the control group. Then, the control probability of a *good* or *very good* outcomes is expected to be 0 . 7. Investigators assume the common odds ratio of the cumulative distributions and wish to achieve the probability 0 . 85 of a *good* or *very good* outcomes to test the effect of the experimental treatment. This provides the reference improvement 0 . 887 for superiority of the experimental treatment and the response probability for the experimental treatment [14]. Table 2 describes the null and alternative probabilities that patients’ response falls in each category. We consider target error rates *α* = 0 . 05 and *β* = 0 . 1 for the test.

**Table 2:** Response probability $p_{ij},i=1,2,j=1,2,3,4$, which denotes the probability that an ordinal outcome on treatment *i* falls in the *j*th level.

	Very good (*j* = 1)	Good (*j* = 2)	Moderate (*j* = 3)	Poor (*j* = 4)
$p_{1j}$	0.2	0.5	0.2	0.1
$p_{2j}$	0.378	0.472	0.106	0.044

Table 3 summarizes the decision rules of F design, FS design, and 1 design. We investigate the operating characteristics of the proposed design through simulations with 10000 replications. We observe that all designs preserve the overall type I and II error rates at target levels. Similarly to Example 1, Criterion 1 yields a smaller size for the first stage and results in the minimum value of EN0 among the criteria. In all decision rules for two-stage designs in Table 3, the maximum sample size of the trial is almost the same.

**Table 3:** Decision rule and its operating characteristics such as overall type I error rate denoted by $\hat{\alpha }$, power, expected sample size under null hypothesis denoted by EN0, and expected sample size under alternative hypothesis denoted by ENa. The sample size $n_{1}$ and $n_{2}$ are obtained by rounding up the results.

		Decision rule					Operating characteristics			
		$t_{1}$		$n_{1}$	$t_{2}$	$n_{2}$	$\hat{\alpha }$	Power	EN0	ENa
F design	Criterion 1	0.048		37	0.126	98	0.041	0.915	116.21	189.88
F design	Criterion 2	-5.711		1	0.126	98	0.047	0.948	196	196
F design	Criterion 3	0.043		35	0.126	98	0.040	0.917	115.70	190.29
		Decision rule					Operating characteristics			
		$v_{1}$	$v_{2}$	$n_{1}$	$v_{3}$	$n_{2}$	$\hat{\alpha }$	Power	EN0	ENa
FS design	Criterion 1	0.046	0.393	36	0.126	99	0.040	0.917	116.84	177.12
FS design	Criterion 2	-0.012	0.234	43	0.147	97	0.043	0.917	142.83	133.02
FS design	Criterion 3	0.035	0.255	42	0.138	98	0.040	0.918	124.50	139.31
		Decision rule					Operating characteristics			
		$t_{1}$		$n_{1}$	$t_{2}$	$n_{2}$	$\hat{\alpha }$	Power	EN0	ENa
1 design		-*∞*		78	0.141	78	0.048	0.897	156	156

The common odds ratio assumption for the cumulative distributions holds in this example. Using the proportional odds model, Whitehead (1993) [14] proposes a method for determining the sample size of the trial based on the score test. The method of Whitehead (1993) [14] requires a total sample size of 188 (i.e., 94 patients per group) to achieve the improvement of the experimental treatment for the trial with a type I error rate of 0.05 and power 90%. This is obtained based on a single-stage test. We notice that the difference in the required total sample size of the trial is very small, but the proposed two-stage designs use, on average, 52.65 fewer patients across the six decision rules compared to the single-stage design in the method of Whitehead (1993) [14], when the experimental treatment is not effective. Moreover, the proposed single-stage design using the Mann-Whitney-Wilcoxon test requires 36 fewer patients in total compared to using the score test.

## Trial illustration

The trial NCT03183167 is a longitudinal cohort study for patients with intracerebral hemorrhage (ICH), which is a devastating sub-type of stroke with a high mortality rate. Hagen et al. (2019) [27] analyzes the study data collected from 2008 to 2015 in order to investigate whether systematic inflammatory response syndrome (SIRS) is predictive of a long-term functional outcome in patients with spontaneous ICH. Functional outcome is evaluated using the modified Rankin Scale (mRS) at 3 and 12 months. The mRS is a widely used measure for assessing functional disability and dependence in individuals who have experienced stroke or other neurological conditions. The ordinal form of mRS is more strongly related to the 5-year mortality rate than the dichotomous mRS, which splits the mRS into favorable or unfavorable outcomes (e.g., using 0-1/2-6 or 0-2/3-6 dichotomies) [13]. To compare patients with noninfectious SIRS to a control group of patients without systematic inflammatory response, Hagen et al. (2019) [27] use propensity score matching to have a well-balanced cohort of 104 patients per group [27]. The mRS at 3 months for patients with SIRS falls in categories 0 to 6 with probabilities 0.048, 0.077, 0.173, 0.125, 0.202, 0.125, 0.25 while the mRS at 3 months for patients in the control group falls in categories 0 to 6 with probabilities 0.058, 0.154, 0.25, 0.163, 0.154, 0.106, 0.115.

Using the information, we apply the proposed designs to determine the monitoring rule and sample size for the trial. Since investigators argue that SIRS is associated with poor mRS, the alternative hypothesis has the opposite inequality sign to our description, i.e., $H_{a}:F(y)>G(y)$. Suppose that target error rates are *α* = 0 . 05 and *β* = 0 . 2. By applying Eqs (4)–(7) and (8)–(12), decision rules are obtained for F design and FS design. We also add the decision rule for 1 design. The performance is evaluated based on 10000 replications through simulation. The results are summarized in Table 4. The F design using Criterion 1 determines $t_{1}=0.064,n_{1}=30,t_{2}=0.133,n_{2}=98$. The decision rule yields a power of 83% and is expected to use 105.17 patients when SIRS is not predictive of poorer long-term functional outcome. The FS design using Criterion 1 determines $v_{1}=0.065,v_{2}=0.452,n_{1}=31,v_{3}=0.134,n_{2}=99$. The decision rule yields a power of 83.8% and is expected to use 106.95 patients when SIRS is not predictive of poorer long-term functional outcome. The F and FS designs with Criterion 1 show that overall error rates are controlled at target levels and spend only 30% of the trial’s information to make a decision earlier than others. Notably, the FS design using Criterion 2 or 3 requires a total sample size of less than 80 patients per group, which is smaller than other decision rules for two-stage designs. This decision rule would be preferable if investigators want to minimize the maximum sample size for the trial.

**Table 4:** Decision rule and its operating characteristics such as overall type I error rate denoted by $\hat{\alpha }$, power, expected sample size under null hypothesis denoted by EN0, and expected sample size under alternative hypothesis denoted by ENa.

		Decision rule					Operating characteristics			
		$t_{1}$		$n_{1}$	$t_{2}$	$n_{2}$	$\hat{\alpha }$	Power	EN0	ENa
F design	Criterion 1	0.064		30	0.133	98	0.039	0.830	105.17	180.65
F design	Criterion 2	-17.45		1	0.136	95	0.049	0.902	190	190
F design	Criterion 3	0.050		29	0.136	95	0.038	0.836	106.21	176.92
		Decision rule					Operating characteristics			
		$v_{1}$	$v_{2}$	$n_{1}$	$v_{3}$	$n_{2}$	$\hat{\alpha }$	Power	EN0	ENa
FS design	Criterion 1	0.065	0.452	31	0.134	99	0.038	0.838	106.95	174.99
FS design	Criterion 2	-0.051	0.290	39	0.161	78	0.044	0.818	128.36	128.27
FS design	Criterion 3	-0.006	0.337	36	0.155	79	0.043	0.822	115.83	136.19
		Decision rule					Operating characteristics			
		$t_{1}$		$n_{1}$	$t_{2}$	$n_{2}$	$\hat{\alpha }$	Power	EN0	ENa
1 design		-*∞*		69	0.160	69	0.050	0.802	138	138

## Discussion

We have proposed optimal two-stage clinical trial designs for ordinal data using the Mann-Whitney-Wilcoxon test. The proposed designs enable monitoring treatment efficacy during the trial and making an efficient decision earlier due to futility or superiority based on interim data. The proposed design works with three criteria depending on the objective of optimality. The optimal two-stage design with Criterion 1 minimizes the expected total sample size when the experimental treatment is not efficacious compared to the control. The optimal two-stage design with Criterion 2 minimizes the expected total sample size when the experimental treatment is efficacious compared to the control. The optimal two-stage design with Criterion 3 minimizes the expected overall total sample size. We provide explicit formulas for calculating sample sizes and identifying stopping boundaries for two-stage designs. The operating characteristics of the proposed designs are investigated through simulations. The simulation results show that overall type I and II error rates are well controlled at target rates. The proposed designs do not require a proportional odds assumption for the ordinal data and work well regardless of the proportional odds assumption. Thus, the proposed designs offer more options for clinical practitioners to consider when designing randomized clinical trials whose primary endpoint is an ordinal outcome.

In this study, we do not employ sample size adaptation. This choice aligns with our focus on a fixed sample size framework, which facilitates a more straightforward analysis and application of the proposed method. Optimizing the adaptation rule introduces additional complexities that are beyond the scope of this work. However, this is an area of interest for future research, where re-estimating the sample size at interim analysis could enhance the flexibility of clinical trial designs.

We have assumed that the primary ordinal data would be evaluated at fixed timelines and fully observed before the interim analysis. However, missing data in clinical trials is almost unavoidable due to factors such as patient dropout, loss of follow-up, or technical issues during data collection. For example, a COVID-19 patient discharged early may not have complete follow-up data for subsequent ordinal outcomes, such as disease progression. Additionally, clinical trials for immunotherapies, where responses can take weeks or months to manifest, and chronic conditions, where outcomes like organ failure or survival may occur long after the intervention, often encounter late-onset outcomes. These delayed events may remain unobserved by the end of the trial, resulting in incomplete ordinal outcome data. This presents opportunities to enhance the proposed method by incorporating advanced statistical approaches, such as multiple imputation or joint modeling, to address missing ordinal data effectively.
